# Potential cerebrospinal fluid metabolomic biomarkers and early prediction model for Parkinson’s disease

**DOI:** 10.3389/fnagi.2025.1582362

**Published:** 2025-05-30

**Authors:** Yifan Zhang, Yuexin Yan, Xiangxu Kong, Haijun Zhang, Shengyuan Su

**Affiliations:** ^1^Department of Intensive Care Medicine, Shenzhen Baoan People’s Hospital, Shenzhen, China; ^2^Department of Neurology, Shenzhen Baoan People’s Hospital, Shenzhen, China

**Keywords:** Parkinson diseases, metabolomic biomarkers, early prediction model, bidirectional Mendelian randomization, PD susceptibility

## Abstract

**Objective:**

To identify key cerebrospinal fluid (CSF) metabolomic biomarkers associated with Parkinson’s disease (PD) and prodromal PD, providing insights for intervention strategy development.

**Methods:**

Six hundred and thirty-nine participants from the Parkinson’s Progression Markers Initiative (PPMI) cohort were included: 300 PD patients, 112 healthy controls (HC), and 227 prodromal PD patients. Regression analysis and OPLS-DA identified metabolic biomarkers, while pathway analysis examined their relationship to clinical features. An XGBoost-based early prediction model was developed to assess the distinction between PD, prodromal PD, and HC. A two-sample bidirectional Mendelian randomization analysis was performed to examine the association between differential metabolites and Parkinson’s disease.

**Results:**

Sixty-four metabolites were significantly altered in PD patients compared to HC, with 58 elevated and 6 reduced. In prodromal PD, 19 metabolites were increased, and 34 were decreased. Key metabolic pathways involved glutathione and amino acid metabolism. Dopamine 3-O-sulfate correlated with PD progression, levodopa-equivalent dose, and non-motor symptoms. The XGBoost model demonstrated high specificity in predicting the onset of PD. The MR analysis results showed a positive correlation between higher genetic predictions of dopamine 3-O-sulfate levels and the risk of Parkinson’s disease. In contrast, the reverse MR analysis found that Parkinson’s disease susceptibility significantly increased dopamine 3-O-sulfate levels.

**Conclusion:**

The differential expression of CSF metabolites reveals early cellular metabolic changes, providing insights for early diagnosis and monitoring PD progression. A bidirectional causal relationship exists between genetically determined PD susceptibility and metabolites.

## Introduction

Parkinson’s disease (PD) is a motor disorder characterized by the progressive loss of dopaminergic neurons in the substantia nigra and the abnormal aggregation of α-synuclein. The motor symptoms of PD are primarily tremor, bradykinesia, and rigidity. In addition to these motor deficits, PD also encompasses a range of non-motor and prodromal symptoms, such as sleep disturbances, olfactory dysfunction, psychiatric and mood changes, cognitive impairment, and autonomic dysfunction. These non-motor symptoms may appear either before or concurrently with the onset of motor symptoms (Marinus et al., 2018).

Although numerous hypotheses regarding the pathogenesis of PD have been proposed, there is currently no effective method to slow the progression of the disease. This complexity arises from the involvement of multiple brain regions and various neurotransmitter systems. These systems include the co-release of dopamine, a typical neurotransmitter, and other excitatory or inhibitory neurotransmitters, which contribute to clinical heterogeneity (Barcomb and Ford, 2023). While the accuracy of clinical diagnosis has improved in the past decade, especially in the early stages, reaching up to 90.3% in some studies (Virameteekul et al., 2023), predicting disease progression in the early stages remains challenging. This is primarily due to the overlap of early clinical features, the complexity of disease subtypes, and the limitations of diagnostic criteria (Tolosa et al., 2021). The prodromal phase is considered a critical window for intervention, making early and accurate diagnosis essential. Predicting disease progression based on reliable and sensitive early biomarkers and quantifying different pathological states of PD remain key research focuses (Theis et al., 2024).

Early diagnosis of PD primarily involves clinical symptom assessment, biochemical testing, imaging techniques, and genetic analysis (Parab et al., 2023; Mitchell et al., 2021). Cerebrospinal fluid (CSF), which directly interacts with brain cells, offers an accurate reflection of the underlying molecular mechanisms of PD. While the α-synuclein seed amplification assay in CSF demonstrates high sensitivity and specificity, it reflects only part of the disease pathology, highlighting the need for additional biomarkers to fully characterize PD (Postuma and Berg, 2016). CSF metabolomics, through the mapping and quantification of various small-molecule metabolites, provides a comprehensive insight into cellular metabolism and neurotransmitter alterations (Stoessel et al., 2018). With recent advancements in liquid chromatography-mass spectrometry (LC-MS/MS), key biomarkers related to lipid metabolism, polyamines, amino acids, and purine metabolism have garnered increasing attention (Trezzi et al., 2017; Kremer et al., 2021).

This study aims to explore the differences in various metabolites, particularly lipid metabolites, at different clinical stages of PD (including healthy controls, prodromal PD, and clinically diagnosed PD patients) using CSF metabolomics as a data-driven source. The study further aims to predict the risk of PD progression. The objectives of this study are as follows: (1) to identify cerebrospinal metabolic biomarkers at different stages of PD progression; (2) to assess the reliability of predictive models by developing a clinical risk model for PD; (3) to link lipid metabolism biomarkers with clinical manifestations to provide clinical utility; (4) to uncover potential mechanisms underlying PD progression through metabolic biomarkers and associated molecular pathways; (5) MR analysis was conducted using publicly available genome-wide association data to evaluate the causal relationship between differential metabolites and Parkinson’s disease.

## Materials and methods

### Study participants

This study utilized data from the Parkinson’s Progression Markers Initiative (PPMI) database, a large-scale clinical observational study aimed at identifying biomarkers of PD progression from the prodromal phase through to disease onset. A total of 639 participants were included in the analysis, with data collection completed by January 2020. The sample size was determined based on previous studies (Huntwork-Rodriguez et al., 2023). Participants were classified into three groups based on predefined inclusion criteria: (1) PD patients: individuals diagnosed with PD, who were undergoing levodopa treatment. (2) Healthy controls: individuals with no history of neurological disorders, no first-degree family history of PD, and normal dopamine transporter (DAT) single-photon emission computed tomography (SPECT) imaging. (3) Prodromal participants: individuals who had not been clinically diagnosed with PD but exhibited one or more of the following risk factors: rapid eye movement sleep behavior disorder (RBD), olfactory dysfunction, dopamine transporter (DAT) deficiency, or genetic variants associated with an increased risk of PD. The prodromal cohort has as inclusion criteria age >60 years (with the exception of SCNA and other rare mutations).

Baseline demographic information, motor and non-motor assessments, and biochemical test results were collected for all participants. All participants underwent lumbar puncture for CSF collection, followed by metabolite and lipid analysis. Data for this study were accessed via the PPMI online database.^1^ The PPMI study received ethical approval from the institutional review boards of over 50 research centers globally. Detailed information regarding the ethics committees of the clinical centers can be found in Supplementary Table 1. All participants provided written informed consent before inclusion in the study, in accordance with ethical guidelines (Brumm et al., 2023). The methodology of this study complies with the relevant guidelines of the PPMI Data and Publications Committee (DPC), and the manuscript was submitted to the DPC for review. Genetic association summary data were obtained from GWAS, with dopamine 3-O-sulfate GWAS data sourced from the Wisconsin Alzheimer’s Disease Research Center (WADRC) and the Wisconsin Registry for Alzheimer’s Prevention (WRAP) cohorts, two European populations (Panyard et al., 2021). The data included 412 cerebrospinal fluid metabolites from 291 samples. Parkinson’s disease GWAS data were sourced from the International Parkinson’s Disease Genomics Consortium (Nalls et al., 2019), comprising 33,674 PD cases and 449,056 control samples.

### Cerebrospinal fluid metabolite and lipid analysis

CSF samples collected from participants were analyzed using liquid chromatography–tandem mass spectrometry (LC-MS/MS), employing both targeted metabolomics/lipidomics and untargeted metabolomics approaches. A total of 348 compounds were identified, including sphingolipids, polyamines, cholesterol, gangliosides, ceramides, amino acids, caffeine, and purine metabolites. To minimize batch effects, internal references were established separately for PD patients and healthy controls prior to analysis. The primary outcome measure was the batch-normalized area ratio for each compound, quantified as the adjusted area ratio.

### Data processing and analysis

Descriptive statistical analysis and intergroup comparisons were performed for demographic data and clinical assessment parameters. For CSF metabolite area ratio data, log10 transformation, mean centering, and scaling normalization were performed using MetaboAnalystR 6.0. Statistical differences in metabolite concentrations between groups were assessed using fold-change analysis and unpaired *t*-tests (or paired *t*-tests, as applicable). Bonferroni correction was applied to adjust the *p*-values for multiple comparisons, ensuring that the family-wise error rate was controlled. Orthogonal partial least squares discriminant analysis (OPLS-DA) was used for enhancing the differentiation between PD, healthy control, and prodromal groups.

Metabolites were considered significantly different if they met the following criteria: fold-change, Bonferroni-corrected *p*-value <0.05, and variable importance in projection (VIP) score >1. Differential metabolites between PD, healthy HC, and prodromal groups were subsequently identified and mapped to the Small Molecule Pathway Database (SMPDB), Relational database of Metabolomic Pathways (RaMP-DB), and Kyoto Encyclopedia of Genes and Genomes (KEGG) pathway databases for enrichment analysis to identify relevant biological pathways. From the list of differential metabolites, the top 8–15 compounds, based on VIP values, were selected for machine learning models. The dataset was randomly divided into a training set (70%) and a validation set (30%). A predictive model was constructed using the XGBoost algorithm to assess PD risk and the ability to distinguish PD from the prodromal phase. The SHapley Additive exPlanations (SHAP) method was applied to provide interpretable explanations for the model’s predictions, elucidating the contribution of each feature to the model outcomes. Furthermore, Spearman’s correlation tests were performed to assess the relationships between the identified differential metabolites and various clinical evaluation parameters in both PD and healthy control groups.

MR analysis included SNPs closely related to the exposure, with significance set at *p* < 5 × 10^−5^ and a linkage disequilibrium threshold of *r*^2^ < 0.001. Sensitivity analysis was performed using inverse variance weighted (IVW) method, weighted median (WM), and MR-Egger regression methods. False discovery rate (FDR) was used to control the adjusted significance levels. Horizontal pleiotropy was tested using the MR-Egger intercept test and MR pleiotropy residual sum and outlier (MR-PRESSO) analysis. Cochran’s *Q* test was used to assess heterogeneity in causal estimates for each SNP. All statistical analyses were conducted using R version 4.4.2.

## Results

### Baseline descriptive characteristics

This study included 300 PD patients, 112 HC, and 227 prodromal individuals. The demographic characteristics of each group are summarized in Table 1. No significant differences were observed among the three groups in terms of age, sex, handedness, or body mass index (BMI). The majority of participants were Caucasian, with four healthy controls having a secondary family history of Parkinson’s disease. Interestingly, prodromal individuals had a significantly higher level of education compared to the HC. This may reflect increased health awareness in individuals with higher education, potentially leading to earlier detection of abnormal symptoms. Compared to HC, prodromal individuals exhibited more significant cognitive impairments, depression, anxiety, and autonomic dysfunction. Among PD patients, most were classified as stage 2 on the Hoehn and Yahr (HY) scale.

**Table 1:** Demographic and clinical characteristics of PD patients, healthy controls, and prodromal individuals.

Demographic or clinical characteristics	Group			*p*-value		
	PD (*n* = 300)	HC (*n* = 112)	Prodromal (*n* = 227)	PD vs. HC	PD vs. prodromal	Prodromal vs. HC
Demographics						
Age (years)	64.55 [57.81, 70.18]	63.63 [57.44, 69.36]	63.84 [58.34, 69.00]	0.475	0.578	0.710
Gender (male) (%)	170 (56.67)	61 (54.46)	124 (54.63)	0.7724	0.705	0.907
Race				0.1576	0.0716	0.0185
White	276.00 (92.00)	105.00 (93.75)	218.00 (96.04)			
Black	6.00 (2.00)	5.00 (4.46)	1.00 (0.44)			
Asian	4.00 (1.33)	1.00 (0.89)	2 (0.88)			
Other (includes multi-racial)	14.00 (4.67)	1.00 (0.89)	6.00 (2.64)			
Fampd bin	122.00 (40.67)	4.00 (3.57)	194.00 (85.46)	<0.001	<0.001	<0.001
Handed						
Right	261.00 (87.00)	91.00 (81.25)	191.00 (84.14)			
Left	29.00 (9.67)	14.00 (12.50)	26.00 (11.45)			
Mixed	10.00 (3.33)	7.00 (6.25)	10 (4.4)			
BMI (kg/m^2^)	26.56 [23.81, 29.72]	26.05 [23.69, 29.59]	26.66 [24.42, 29.75]	0.630	0.128	0.099
Education						
EDUCYRS	16.00 [14.00, 18.00]	16.00 [13.00, 17.25]	18.00 [16.00, 19.00]	0.121	<0.001	<0.001
Duration	1.09 [0.40, 2.83]					
sym_tremor	230.00 (76.67)					
sym_rigid	209.00 (69.67)					
sym_brady	209.00 (69.67)					
sym_posins	38.00 (12.67)					
sym_other	46.00 (15.33)					
PDTRTMNT	197.00 (65.67)					
LEDD	300.00 [0.00, 599.25]					
Non-motor assessments						
MOCA score	27.00 [25.00, 29.00]	28.00 [27.00, 29.00]	27.00 [25.00, 29.00]	<0.001	0.596	<0.001
GDS	2.00 [1.00, 4.00]	1.00 [0.00, 2.00]	1.00 [0.00, 3.00]	<0.001	<0.001	0.002
STAI	65.00 [52.00, 80.00]	54.00 [44.75, 65.00]	54.00 [46.00, 68.25]	<0.001	<0.001	0.149
STAI_state	32.00 [25.00, 41.00]	25.00 [21.00, 32.25]	26.00 [21.00, 32.25]	<0.001	<0.001	0.269
STAI_trait	33.00 [26.00, 39.00]	27.00 [22.75, 33.00]	29.00 [24.00, 37.00]	<0.001	0.001	0.044
SCOPA	11.00 [6.50, 16.00]	5.00 [3.00, 8.50]	7.00 [4.00, 10.25]	<0.001	<0.001	0.002
NP1DPRS				0.008	0.001	0.987
Normal	219.00 (73.00)	96.00 (85.71)	195.00 (85.90)			
Slight	64.00 (21.33)	13.00 (11.61)	18.00 (7.93)			
Mild	9.00 (3.00)	3.00 (2.68)	9.00 (3.96)			
Moderate	5.00 (1.67)	0.00 (0.00)	4.00 (1.76)			
Severe	3.00 (2.68)	0.00 (0.00)	1 (0.44)			
NP1ANXS				<0.001	0.003	0.124
Normal	177.00 (59.00)	87.00 (77.68)	159.00 (70.04)			
Slight	91.00 (30.33)	24.00 (21.43)	58.00 (25.55)			
Mild	20.00 (6.67)	1.00 (0.89)	8.00 (3.52)			
Moderate	9.00 (3.00)	0.00 (0.00)	1.00 (0.44)			
Severe	3.00 (2.68)	0.00 (0.00)	1.00 (0.44)			
Motor assessments						
NHY staging	1 [2, 2]					
MDS-UPDRS I score	7.00 [4.00, 10.00]					
MDS-UPDRS II score	6.00 [4.00, 10.00]					
MDS-UPDRS III score (OFF)	24.00 [17.00, 31.00]					
MDS-UPDRS IV score (OFF)	0.00 [0.00, 3.00]					
MDS-UPDRS total score (OFF)	37.00 [27.00, 50.00]					

Non-normally distributed continuous variables are presented as median [quartiles], categorical variables are presented as number (percentage). GDS, Geriatric Depression Scale score; STAI, STAI total score; sym_, initial symptoms; PDTRTMNT, dopa-treated patients; NP1DPRS, MDS-UPDRS Part I Depressed Mood; NP1ANXS, MDS-UPDRS Part I Anxious Mood.

### Cerebrospinal fluid metabolite analysis

**Figure 1:**
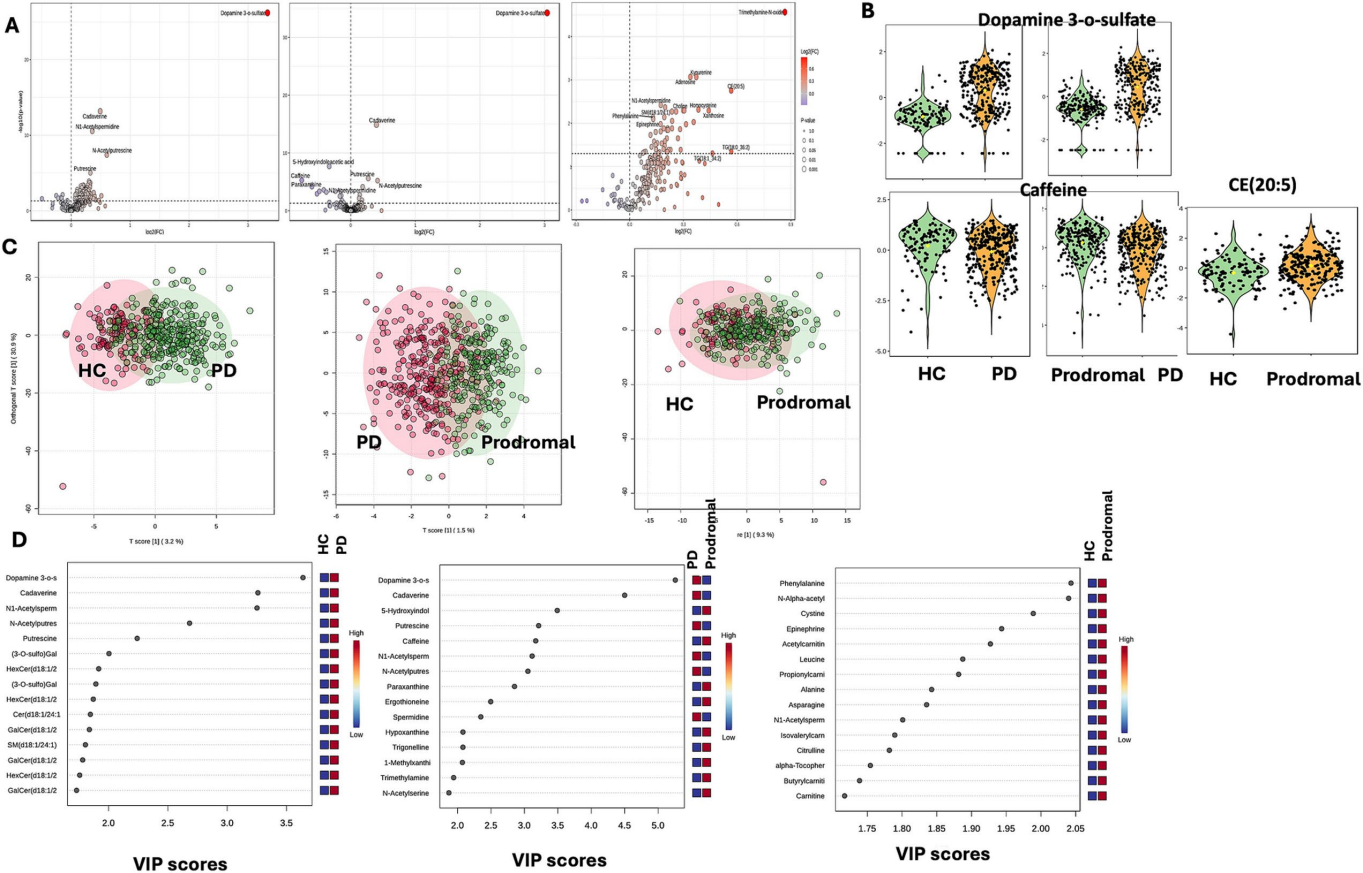
Univariate and multivariate metabolomic analysis. Metabolomic profiling. **(A)** Volcano plots showing differential metabolites between PD and HC, highlighting dopamine 3-O-sulfate and timethylamine. **(B)** Violin plots depicting key metabolites [dopamine 3-O-sulfate, caffeine, CE (20:5)] in HC, PD, and prodromal groups. **(C)** OPLS-DA score plots illustrating group separation based on metabolic profiles. **(D)** VIP score plots for top differential metabolites between groups.

To preliminarily identify differential metabolites, metabolites were selected based on adjusted *p*-values (after *t*-test analysis) and VIP scores greater than 1.0 (from OPLS-DA model calculations). Compared to HCs PD patients showed significant differences in 64 metabolites, of which 58 were increased and 6 were decreased. Compared to prodromal individuals, PD patients exhibited 19 metabolites with increased levels and 34 metabolites with significantly reduced levels. Notably, dopamine 3-O-sulfate was significantly elevated in PD patients (PD vs. HC: FC = 10.066, *p*-adjust <0.001, VIP = 3.638; PD vs. prodromal: FC = 3.039, *p*-adjust <0.001, VIP = 5.257), while caffeine levels were decreased (PD vs. HC: FC = 0.709, *p*-adjust = 0.026, VIP = 1.663; PD vs. prodromal: FC = 0.591, *p*-adjust <0.001, VIP = 3.169). Additionally, compared to HC, prodromal individuals showed increased levels in 41 metabolites, with CE (20:5) being higher in the prodromal group (FC = 1.481, *p*-adjust = 0.002, VIP = 1.343). Figure 1 illustrates the visual results of univariate and multivariate statistical analyses, and Table 2 provides a detailed list of the top 15 core differential metabolites ranked by VIP scores (8 metabolites are shown due to the limited number of differential metabolites between PD and prodromal groups).

**Table 2:** Differential metabolomic analysis of cerebrospinal fluid among Parkinson’s disease (PD) patients, healthy controls (HC), and prodromal individuals.

Group	Compound name	VIP value	Fold change	*p* adjust
PD vs. HC	Dopamine 3-o-sulfate	3.638	10.066	<0.001
	Cadaverine	3.258	1.406	<0.001
	N1-Acetylspermidine	3.251	1.281	<0.001
	N-Acetylputrescine	2.683	1.519	<0.001
	Putrescine	2.241	1.253	<0.001
	(3-O-sulfo)GalCer(d18:1/24:1)	2.004	1.232	<0.001
	HexCer(d18:1/24:1)	1.917	1.236	<0.001
	(3-O-sulfo)GalCer(d18:1/24:0)	1.893	1.253	<0.001
	HexCer(d18:1/24:0)	1.871	1.250	<0.001
	Cer(d18:1/24:1)	1.847	1.234	0.002
	GalCer(d18:1/24:1)	1.839	1.214	<0.001
	SM(d18:1/24:1)	1.805	1.196	0.001
	GalCer(d18:1/20:0)	1.782	1.192	0.001
	HexCer(d18:1/22:0)	1.757	1.221	0.002
	GalCer(d18:1/24:0)	1.731	1.204	0.001
	Cadaverine	4.499	1.318	<0.001
	Dopamine 3-o-sulfate	5.257	8.221	<0.001
	Homoserine	1.809	1.101	0.002
PD vs. prodromal	N-Acetylputrescine	3.052	1.333	<0.001
	N1-Acetylspermidine	3.115	1.136	<0.001
	Putrescine	3.213	1.207	<0.001
	Spermidine	2.348	1.128	0.014
	Threonine	1.683	1.094	0.007
	(3-O-sulfo)GalCer(d18:1/24:1)	1.214	1.141	0.013
	alpha-Tocopherol	1.754	1.281	0.009
	Butyrylcarnitine	1.739	1.099	0.026
	CE(18:2)	1.471	1.171	0.041
	CE(20:5)	1.343	1.481	0.002
	Cer(d18:1/24:1)	1.158	1.196	0.005
	Choline	1.253	1.230	0.005
Prodromal vs. HC	Citrulline	1.782	1.145	0.033
	Cystine	1.989	1.177	0.005
	DG(16:0_18:1)	1.055	1.218	0.041
	DHA	1.128	1.178	0.013
	Dimethylglycine	1.439	1.122	0.046
	Epinephrine	1.943	1.095	0.008
	GalCer(d18:1/16:0)	1.409	1.143	0.007
	GalCer(d18:1/24:1)	1.279	1.155	0.014

VIP, variable importance in projection; FC, fold change; HexCer, hexosylceramide; SM, sphingomyelin; Cer, ceramide; GalCer, galactosylceramide; CE, cholesteryl ester; DHA, docosahexaenoic acid; DG, diacylglycerol.

### Metabolic pathway analysis

**Figure 2:**
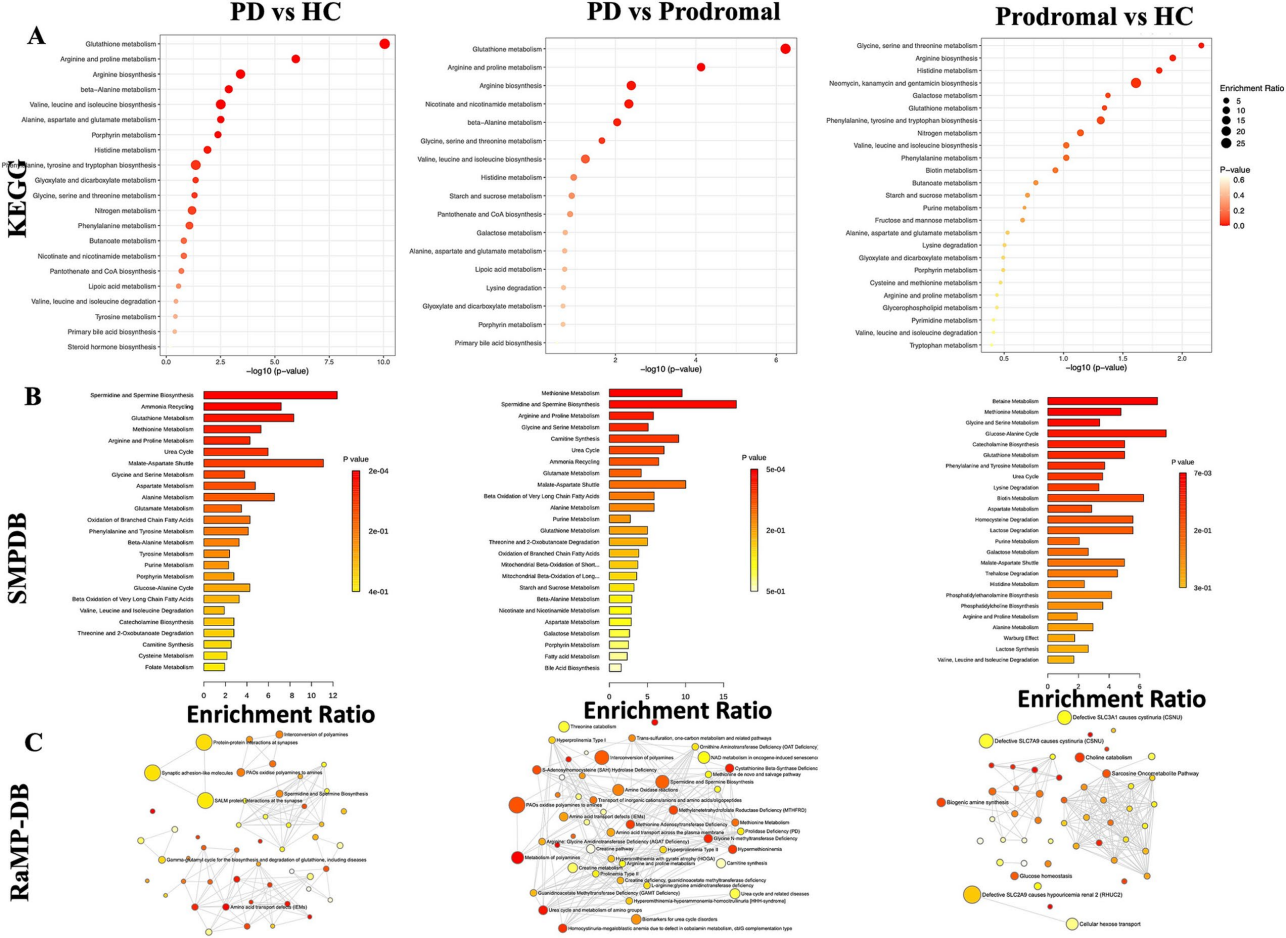
Metabolic pathway enrichment analysis. **(A)** KEGG pathway enrichment showing key pathways, such as glutathione and amino acid metabolism, across PD, prodromal PD, and HC groups. **(B)** SMDB pathway analysis highlighting metabolic disruptions in amino acid and steroid metabolism. **(C)** RaMP-DB network illustrating the connection between altered pathways and neurodegenerative diseases.

We performed pathway enrichment analysis using the KEGG database for the core differential metabolites across the PD, HC, and prodromal groups. Compared to HC and prodromal individuals, the most significantly regulated pathways in the CSF of PD patients were metabolism of glutathione, metabolism of arginine and proline, and the biosynthesis of arginine. Additionally, compared to HC, prodromal individuals showed the highest enrichment levels in glycine, serine, and threonine metabolism. A total of 13 shared pathways were identified across the PD, HC, and prodromal groups, primarily involving amino acid metabolism. Further annotation using the SMPDB database confirmed these findings, highlighting nine shared pathways, including glutathione metabolism, amino acid metabolism, methionine metabolism, purine metabolism, and the urea cycle (Figure 2). Figure 2C, based on RaMP-DB annotations, presents biological and lipid metabolism pathways. Specifically, PD was associated with synaptic interaction pathways and polyamine oxidation processes, while prodromal individuals showed changes in choline metabolism, glucose metabolism, and creatine metabolism pathways.

### Clinical relevance and risk prediction

**Figure 3:**
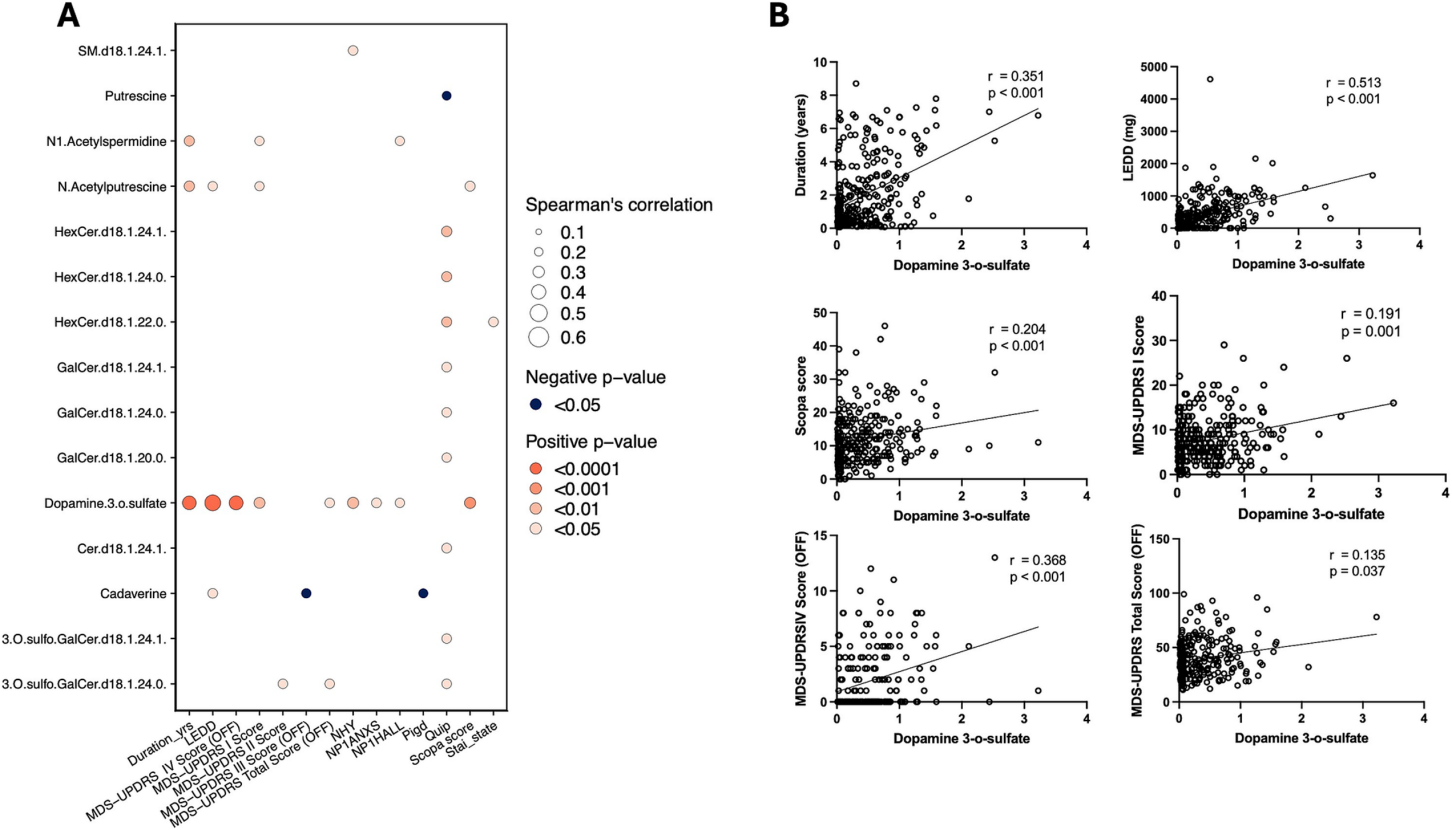
Metabolite-clinical correlation analysis. **(A)** Spearman’s correlation analysis of dopamine 3-O-sulfate with clinical measures, showing significant positive correlations with various PD progression indicators. **(B)** Scatter plots illustrating the correlation between dopamine 3-O-sulfate levels and clinical scores, including disease duration, SCOPA, LEED, and MDS-UPDRS.

We analyzed the association of the 15 core differential metabolites between PD and HC with clinical assessments of PD patients. Dopamine 3-O-sulfate, which was significantly elevated in PD patients’ CSF, showed positive correlations with several clinical parameters, including disease duration, levodopa equivalent daily dose (LEDD), the first and fourth sections of the Unified Parkinson’s Disease Rating Scale (UPDRS), the total score during the “off” period, and The Scale for Outcomes in Parkinson’s disease for Autonomic symptoms (SCOPA-AUT) scores (Figure 3).

**Figure 4:**
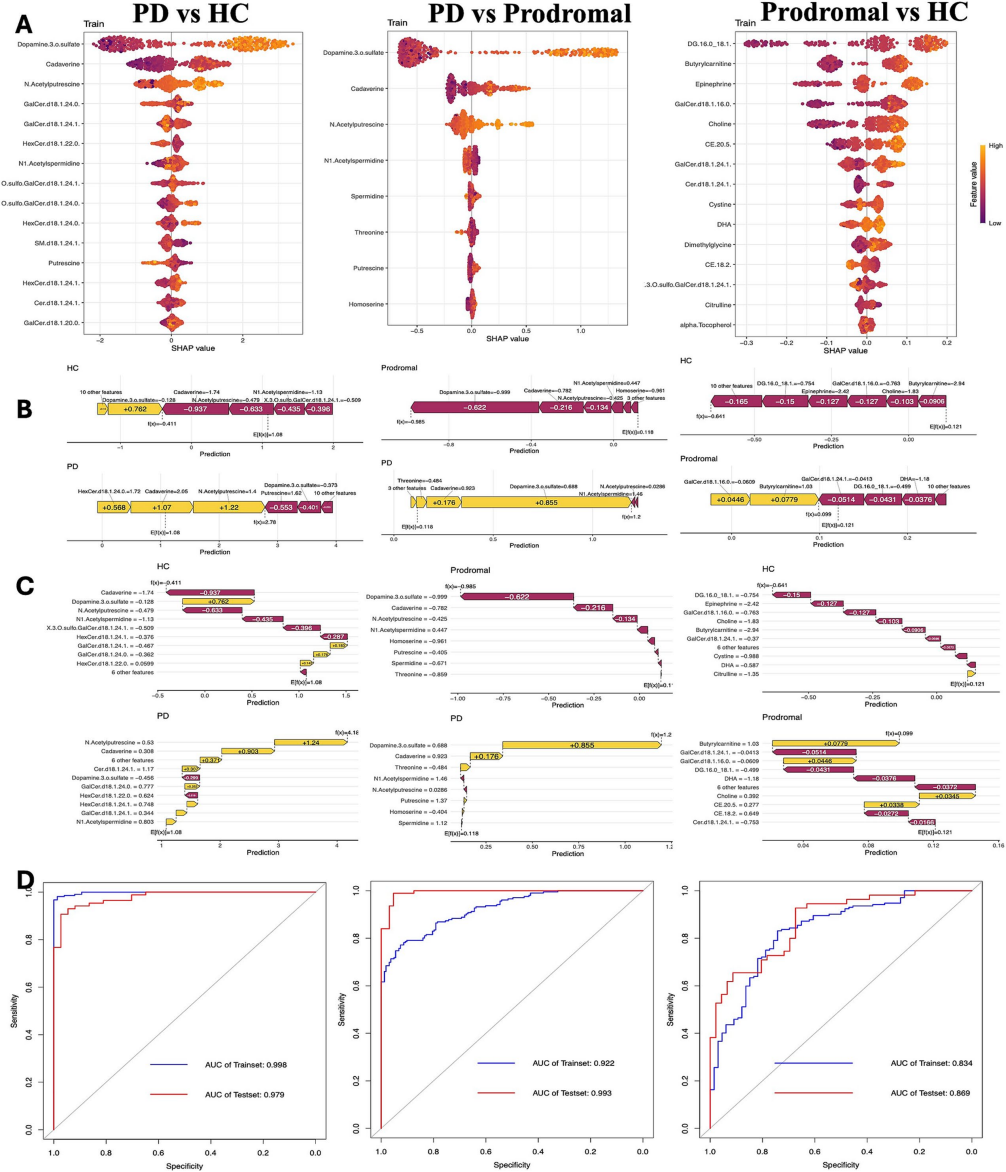
Clinical prediction model analysis. **(A)** Beeswarm plot of SHAP values showing the contribution of key metabolites in distinguishing between PD, prodromal PD, and HC groups. **(B,C)** Feature importance plots for prediction accuracy in PD, prodromal, and HC groups, highlighting the significant metabolites influencing model predictions. **(D)** Receiver operating characteristic (ROC) curves and AUC values assessing the predictive performance of the model in the training and test datasets.

To evaluate the risk of progression from HC to PD and from prodromal to PD, XGBoost models were developed using both training and validation datasets. These models performed excellently, with the area under the curve (AUC) exceeding 0.9 in both datasets. However, the model’s generalization ability from HC to prodromal was slightly lower, with an AUC of 0.834 for the training set and 0.869 for the testing set. The SHapley Additive exPlanations (SHAP) method was used to interpret the XGBoost model and identify the most important predictors. The SHAP algorithm identified the variables with the greatest impact on the model’s predictions. Figure 4 uses a waterfall plot to illustrate the contribution of various metabolites to PD risk prediction in HC, prodromal, and PD groups. Notably, dopamine 3-O-sulfate had the strongest predictive power for PD risk. Additionally, increased levels of DG (16:0_18:1) were associated with a higher risk of prodromal symptoms, while elevated GalCer.d18.1.24.0 levels appeared to lower the risk of progression to PD.

### The association between dopamine 3-O-sulfate and PD

Forward MR analysis showed a positive correlation between increased dopamine 3-O-sulfate levels and PD risk (OR = 1.14, 95% CI: 1.060–1.225, *p*-adjust = 0.002). Reverse MR also confirmed a causal relationship between PD and increased cerebrospinal fluid dopamine 3-O-sulfate levels (OR = 1.337, 95% CI: 1.038–1.337, *p* = 0.011, *p*-adjust = 0.057) (Supplementary Table 2). MR-Egger regression intercepts indicated no significant directional pleiotropy between the SNPs in both groups, with *p*-values greater than 0.05. Cochran’s *Q* test did not detect any detectable heterogeneity (Supplementary Table 3).

## Discussion

The early stages of PD present a critical window for identifying biomarkers that reflect the evolving pathophysiological mechanisms of the disease. Among these, mitochondrial dysfunction, oxidative stress, and energy metabolism are considered primary contributors, particularly in the interaction between dopaminergic neurons and α-synuclein. The accumulation of α-synuclein may induce oxidative stress in dopaminergic neurons, creating a feedback loop that accelerates disease progression (Lai et al., 2024).

In this study, we employed both targeted and untargeted analytical approaches to investigate metabolic differences across various clinical stages of PD: HC, individuals in the prodromal phase, and diagnosed PD patients. Notably, the differences observed between PD versus HC and PD versus prodromal groups exhibited considerable overlap in core metabolites and metabolic pathways. This finding suggests that as PD progresses to its clinical diagnostic stage, CSF metabolic alterations become more pronounced and stable. Dopamine 3-O-sulfate, an inactive form of endogenous dopamine, is likely to cross the blood-brain barrier (Suominen et al., 2015). Its increased concentration may reflect compensatory mechanisms following dopamine depletion (Le Witt et al., 1992). Caloric restriction has been shown to protect dopaminergic neurons by reducing dopamine 3-O-sulfate levels in the substantia nigra, which could potentially provide therapeutic insights (Green et al., 2018). In agreement with previous studies linking CSF dopamine sulfate levels to motor symptom fluctuations and LEDD in advanced PD, our results showed a significant correlation between dopamine 3-O-sulfate levels and both LEDD and UPDRS scores, supporting the involvement of dopamine metabolism in PD progression. Additionally, a bidirectional causal relationship exists between dopamine 3-O-sulfate levels and genetic susceptibility to Parkinson’s disease (PD), suggesting that cerebrospinal fluid dopamine 3-O-sulfate may serve as a biomarker for both the onset and clinical progression of PD. Furthermore, studies indicate that the key enzyme responsible for its production, dopamine sulfotransferase, is associated with the risk of early-onset PD (Butcher et al., 2018).

Dysregulation of dopamine sulfotransferase may contribute to the abnormal accumulation of dopamine 3-O-sulfate in cerebrospinal fluid. Moreover, enzyme imbalance leading to dopamine metabolism abnormalities may trigger the aggregation of abnormal proteins in PD, as well as oxidative stress and neurotoxicity.

We also observed a marked reduction in caffeine levels in PD patients, a finding consistent with earlier reports of lower serum caffeine levels in PD (Suominen et al., 2015). Interestingly, long-term caffeine consumption has been linked to downregulation of the dopamine transporter, which may reduce PD risk (Saarinen et al., 2024). In addition, CE (20:5), a cholesterol ester, was elevated in the prodromal group compared to healthy controls (HC). This metabolite, as an intermediate product of cholesterol metabolism, may signal neuronal injury and membrane disruption in the central nervous system, highlighting a potential link between early metabolic dysregulation and prodromal PD symptoms (Butcher et al., 2018; Saarinen et al., 2024).

Beyond cholesterol metabolites, other lipid metabolic pathways, including phosphatidylcholine (PC), sphingomyelin (SM), and cholesterol esters (CE), are disrupted in the PD brain (Qiu et al., 2023). Increased α-synuclein levels in the PD brain enhance interactions between cellular membrane lipids and α-synuclein, promoting its binding to synaptic and mitochondrial membranes, which in turn accelerates its aggregation (Gilmozzi et al., 2020). The resulting synaptic dysfunction further exacerbates lipid accumulation, creating a vicious cycle (Erskine et al., 2021). Intracellular lipid accumulation may be caused by impaired mitochondrial β-oxidation or defective lipid autophagy (Viennet et al., 2018). Moreover, lipid homeostasis in CSF correlates with the disease stage and progression of PD (Fernández-Irigoyen et al., 2021).

Metabolic pathway analysis revealed distinct metabolic features across healthy controls, prodromal individuals, and diagnosed PD patients. In the prodromal phase, pathways related to glycine, serine, and threonine metabolism were prominently activated, suggesting disruptions in cellular signaling. Conversely, diagnosed PD patients exhibited significant alterations in glutathione metabolism, as well as arginine and proline metabolism, indicating increased oxidative stress and abnormalities in cell proliferation and apoptosis (Wang et al., 2020).

In terms of clinical risk prediction, we developed an XGBoost model using core differential metabolites to predict PD risk. The model showed excellent predictive performance, particularly for the progression from prodromal symptoms to PD, with an AUC value of 0.993 achieved using only eight metabolite variables. However, the prediction ability between healthy controls and the prodromal group was less robust due to the subtle metabolic differences, indicating the need for further validation in longitudinal cohort studies.

In summary, this study highlights the metabolic changes in cerebrospinal fluid across different stages of early Parkinson’s disease. By identifying reliable omics features from a range of candidate biomarkers, this work contributes to the development of more systematic and comprehensive strategies to assess and predict the progression of PD, considering both the mechanisms of the disease and its potential for early intervention.

## Conclusion

This study underscores the potential of metabolomics in identifying biomarkers associated with the progression of Parkinson’s disease (PD). The findings reveal that early disruptions in the homeostasis of neurotransmitters, lipids, and amino acid metabolites differ prodromal PD patients and those who have manifested motor symptoms. Moreover, alterations in related metabolic pathways also reflect changes in cellular functions. Understanding the mechanisms underlying these metabolic changes is crucial for gaining deeper insights into the early progression of the disease.

## Data Availability

The datasets presented in this study can be found in online repositories. The names of the repository/repositories and accession number(s) can be found in the article/Supplementary material.

## Footnotes

^1^ https://www.ppmi-info.org/access-data-specimens/download-data/
