# Supplementary material for: Potential cerebrospinal fluid metabolomic biomarkers and early prediction model for Parkinson’s disease
Source: Front Aging Neurosci. 2025 May 30;17:1582362. doi: 10.3389/fnagi.2025.1582362 (PMC12163037; doi:10.3389/fnagi.2025.1582362)
Supplement: Supplementary file 1 [file Data_Sheet_1.docx]

**Supplemental table 1.** Detailed information regarding the ethics committees of the clinical centers.

| **Location** | **Organization** | **PI** |
| --- | --- | --- |
| Ann Arbor, MI | University of Michigan | Kelvin Chou |
| Athens, Greece | National and Kapodistrian University of Athens | Leonidas Stefanis |
| Atlanta, GA | Emory University School of Medicine | Stewart Factor |
| Aurora, CO | University of Colorado Denver | Michelle Fullard |
| Baltimore, MD | Johns Hopkins University | Emile Moukheiber |
| Barcelona, Spain | Hospital Clinic de Barcelona | Eduardo Tolosa |
| Birmingham, AL | University of Alabama at Birmingham | Marissa Dean |
| Boca Raton, FL | Parkinson's Disease and Movement Disorders Center of Boca Raton | Stuart Isaacson |
| Boston, MA | Massachusetts General Hospital | Aleksandar Videnovic |
| Boston, MA | Boston University Medical Center | Marie Saint-Hilaire |
| Chicago, IL | Northwestern University | Tanya Simuni |
| Cincinnati, OH | University of Cincinnati | Alberto Espay |
| Cleveland, OH | The Cleveland Clinic | Hubert Fernandez |
| Donostia-San Sebastian, Spain | Hospital Universitario Donostia | Javier Ruiz Martinez |
| Gainesville, FL | University of Florida | Nikolaus McFarland |
| Houston, TX | Baylor College of Medicine | Arjun Tarakad |
| Innsbruck, Austria | Medical University Innsbruck | Werner Poewe |
| Kansas City, KS | University of Kansas Medical Center | Rajesh Pahwa |
| Kassel, Germany | Paracelsus-Elena Klinik Kassel | Brit Mollenhauer |
| La Jolla, CA | University of California, San Diego | Douglas Galasko |
| Lagos, Nigeria | Lagos College of Medicine, University of Lagos | Njideka Okubadejo |
| Las Vegas, NV | Cleveland Clinic Lou Ruvo Center for Brain Health | Zoltan Mari |
| London, United Kingdom | Imperial College London | Yen Tai |
| London, United Kingdom | Wolfson Institute of Population Health | Dr. Alastair Noyce & Dr. Cristina Simonet |
| Los Angeles, CA | Keck School of Medicine of USC | Mark Lew |
| Luebeck, Germany | University of Luebeck | Christine Klein, Norbert Brüggemann |
| Luxembourg, Luxembourg | University of Luxembourg | Rejko Krueger |
| Marburg, Germany | Philipps-University of Marburg | Wolfgang Oertel |
| Montreal, Canada | Montreal Neurological Institute-Hospital | Ron Postuma |
| New Haven, CT | Institute for Neurodegenerative Disorders/XingImaging | Neha Prakash |
| New York, NY | Columbia University Medical Center | Sarah O'Shea |
| New York, NY | Mount Sinai Beth Israel | Katherine Leaver |
| New York, NY | NYU Langone Health | Giulietta Riboldi |
| Newcastle upon Tyne, England | Clinical Ageing Research Unit, Newcastle University | Nicola Pavese, David Ledingham |
| Nijmegen, Netherlands | Radboud University | Bastiaan Bloem |
| Ottawa, Canada | The Ottawa Hospital | Tiago Mestre |
| Oxford, United Kingdom | John Radcliffe Hospital Oxford and Oxford University | Michele Hu |
| Philadelphia, PA | University of Pennsylvania | Nabila Dahodwala |
| Phoenix, AZ | Barrow Neurological Institute | Holly Shill |
| Pittsburgh, PA | University of Pittsburgh | Lana Chahine |
| Portland, OR | Oregon Health and Science University | Penelope Hogarth |
| Rochester, NY | University of Rochester | Ruth Schneider |
| Salerno, Italy | University of Salerno | Paolo Barone |
| San Francisco, CA | University of California, San Francisco | Caroline Tanner |
| Scottsdale, AZ | Mayo Clinic of Arizona | Charles Adler |
| Seattle, WA | VA Puget Sound Health Care System | Shu-Ching Hu |
| Sun City, AZ | Banner Sun Health Research Institute | David Shprecher |
| Tampa, FL | University of South Florida | Robert Hauser |
| Tel Aviv, Israel | Tel Aviv Medical Center | Anat Mirelman |
| Tel Aviv, Israel | Tel Aviv Sourasky Medical Center | Roy Alcalay |
| Toronto, Canada | Toronto Western Hospital | Connie Marras |
| Tübingen, Germany | University of Tuebingen | Kathrin Brockmann |

**Supplemental table 2**: Mendelian randomization (MR) analysis of the relationship between dopamine 3-O-sulfate and and Parkinson's Disease.

| Exposure | Outcome | Method | OR | 95%CI | P value | P adjust |
| --- | --- | --- | --- | --- | --- | --- |
| Dopamine 3-O-sulfate | PD | MR Egger | 1.169 | 1.030-1.328 | 0.031 | 0.052 |
|  |  | Weighted median | 1.120 | 1.015-1.236 | 0.024 | 0.052 |
|  |  | IVW | 1.140 | 1.060-1.225 | < 0.001 | 0.002 |
|  |  | Simple mode | 1.172 | 1.003-1.371 | 0.066 | 0.083 |
|  |  | Weighted mode | 1.079 | 0.930-1.251 | 0.333 | 0.333 |
|  |  |  |  |  |  |  |
| PD | Dopamine 3-O-sulfate | MR Egger | 1.439 | 0.787-1.439 | 0.694 | 0.694 |
|  |  | Weighted median | 1.328 | 0.977-1.328 | 0.096 | 0.241 |
|  |  | IVW | 1.337 | 1.038-1.337 | 0.011 | 0.057 |
|  |  | Simple mode | 1.467 | 0.911-1.467 | 0.257 | 0.364 |
|  |  | Weighted mode | 1.366 | 0.917-1.366 | 0.291 | 0.364 |

**Supplemental table 3**: Sensitivity test of the Mendelian randomization analysis between Dopamine 3-O-sulfate and PD. Abbreviations: IVW, inverse variance weighted; MR, Mendelian randomization; OAA, overall acceleration average; PD, Parkinson's disease.

| **Exposure** | **Outcome** | **Method** | **Heterogeneity test** | | **Horizontal pleiotropy** | | **MR-PRESSO** |
| --- | --- | --- | --- | --- | --- | --- | --- |
|  |  |  | Cochran's Q | *p* | Egger intercept | *p* | *p* of global test |
|  |  | IVW | 3.268 | 0.997 | -0.008 | 0.639 | 0.998 |
| Dopamine 3-O-sulfate | PD | MR-Egger | 3.498 | 0.998 |  |  |  |
|  |  |  |  |  |  |  |  |
| PD | Dopamine 3-O-sulfate | IVW | 2.208 | 0.994 | 0.019 | 0.486 | 0.995 |
|  |  | MR-Egger | 2.732 | 0.993 |  |  |  |
